# Supplementary material for: Safety Evaluation and Population Pharmacokinetics of Camostat Mesylate and Its Major Metabolites Using a Phase I Study
Source: Pharmaceutics. 2023 Sep 21;15(9):2357. doi: 10.3390/pharmaceutics15092357 (PMC10534584; doi:10.3390/pharmaceutics15092357)
Supplement: Supplementary file 1 [file pharmaceutics-15-02357-s001.zip › pharmaceutics-2589145-supplementary.pdf]

pharmaceutics

Article

Supplementary Materials: Safety Evaluation and Population Pharmacokinetics of Camostat mesylate and its major metabolits using Phase I study

Gyan-Young Jim, Hyun-ki Moon, Taeheon Kim, So-hye Yun, Hwi-yeol Yoon, Jang-hee Hong and Dae-Duk Kim

Table S1. Final model PK parameters and bootstrap results.

Parameter (unit)	Estimate	R.S.E (%)	Bootstrap results	5-95% percentile	Description
Fixed effect					
Ka (1/h)	0.757	3.71	0.754	0.697-0.833	Absorption rate
Tlag (h)	0.204	2.75	0.203	0.18900.213	Lag time
Vd1 (L)	237.9	22.8	248.6	175.7-341.0	Volume of distribution of GBPA
CL1	1269	19.7	1685	1112-2273	Clearance of GBPA
Vd2 (L)	174.6	3.58	160.0	122.9-213.9	Volume of distribution of GBA
CL2 (L/h)	103.7	7.41	94.95	71.13-130.0	Clearance of GBA
FRAC	0.184	0.17	0.168	0.117-0.199	Fraction of cleared as GBPA
V_{max}	2.215	23	1.97	1.334-2.723	
K_m	1.192	0.19	1.89		
Between-subject Variability					
Ka	0.109	31.0	0.095	0.053-0.134	
Tlag	0.089	26.2	0.093	0.047-0.158	
V1	0.813	24.1	0.755	0.531-0.988	
CL1	0.629	26.5	0.489	0.286-0.714	
CL2	0.210	26.4	0.180	0.051-0.281	
V_{max}	0.820	19.7	0.739	0.445-1.027	
Corr_ V_{max}_V1	-0.89	8.62	-0.868		
Error model					
Pro_GBPA	0.25	6.66	0.247	0.209-0.288	Proportional residual error for GBPA
Pro_GBA	0.23	6.74	0.227	0.171-0.283	Proportional residual error for GBA

Ka, absorption rate constant; Tlag, lag time; Vd, volume of distribution; CL, clearance; FRAC, fraction; V_{max}, the reaction rate at the state where the enzyme is fully saturated by the substrate; Km, the concentration of a substrate at which half of the maximum velocity; Corr, correlation between random effects; Pro, proportional error model.

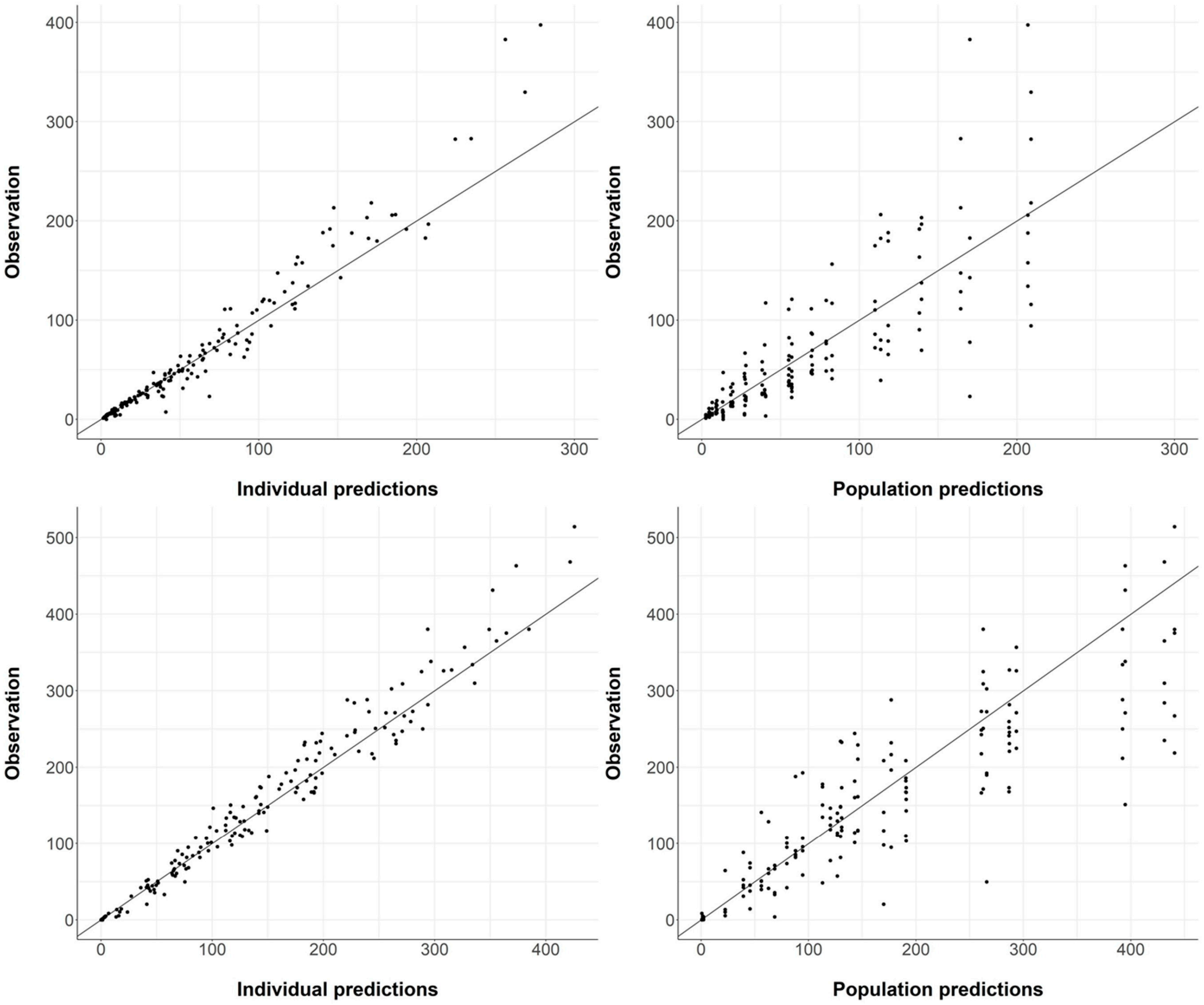

Figure S1. Goodness of fit of GBPA (upper) and GBA (lower). Left is observation versus individual prediction. Right is observation versus population prediction.
